# MRSA in Africa: Filling the Global Map of Antimicrobial Resistance

**DOI:** 10.1371/journal.pone.0068024

**Published:** 2013-07-29

**Authors:** Matthew E. Falagas, Drosos E. Karageorgopoulos, John Leptidis, Ioanna P. Korbila

**Affiliations:** 1 Alfa Institute of Biomedical Sciences (AIBS), Athens, Greece; 2 Department of Internal Medicine-Infectious Diseases, Mitera Hospital, Hygeia Group, Athens, Greece; 3 Department of Medicine, Tufts University School of Medicine, Boston, Massachusetts, United States of America; University Hospital Münster, Germany

## Abstract

We sought to assess the prevalence of methicillin-resistance among *Staphylococcus aureus* isolates in Africa. We included articles published in 2005 or later reporting for the prevalence of MRSA among *S. aureus* clinical isolates. Thirty-two studies were included. In Tunisia, the prevalence of MRSA increased from 16% to 41% between 2002–2007, while in Libya it was 31% in 2007. In South Africa, the prevalence decreased from 36% in 2006 to 24% during 2007–2011. In Botswana, the prevalence varied from 23–44% between 2000–2007. In Algeria and Egypt, the prevalence was 45% and 52% between 2003–2005, respectively. In Nigeria, the prevalence was greater in the northern than the southern part. In Ethiopia and the Ivory Coast, the prevalence was 55% and 39%, respectively. The prevalence of MRSA was lower than 50% in most of the African countries, although it appears to have risen since 2000 in many African countries, except for South Africa.

## Introduction

The example of penicillin-resistant *Streptococcus pneumoniae,* which was first reported in South Africa, in pediatric patients being treated “prophylactically” with antibiotics for serious viral infections, demonstrates the intercalation of different regions of the world, where issues of antibiotic resistance are concerned [1]. Regarding methicillin-resistant *Staphylococcus aureus* (MRSA), another important Gram-positive pathogen, it first emerged in the 1960s, soon after methicillin was introduced into clinical therapeutics, and by the end of that decade it was responsible for hospital outbreaks in Western Europe, Australia, and the United States [2], [3]. In the late 1980s, MRSA outbreaks were noted in Australia among Aboriginal populations without any exposure to hospitals [4], [5]. In the United States and elsewhere, the rates of community-associated (CA)-MRSA were increasing until 2008. Certain risk factors for infection with CA-MRSA have been identified [6], [7], [8]. Recent estimates have reported more people going to hospitals with MRSA infections, than acquiring MRSA infections during hospital stay [9], [10], [11].

The epidemiologic changes in MRSA over the years have shown that the distinction between CA-MRSA and hospital-acquired (HA)-MRSA is no longer clear [12], [13], [14]. By 2004, MRSA had exhibited genetic diversity leading to the emergence of 6 major clones of MRSA worldwide; relating to the Staphylococcal Cassette Chromosome SCCmec types I-VI [15], [16], [17]. These types are spreading even in areas where MRSA did not appear to pose a considerable threat, such as Africa [18], [19], [20]. Particularly in Africa, MRSA clinical isolates have been reported as early as 1978; a hospital outbreak occurred in 1986–1987 at Johannesburg, South Africa, and CA-MRSA infections have been reported since the early 1990s in Zimbabwe [21], [22], [23].

The healthcare systems in Africa as a whole suffer from inadequate financing, shortages in infrastructure, medical equipment and medications, as well as limited supply of adequately trained healthcare professionals (partly related to “brain drain” to developed countries) [24]. The wide spread of communicable diseases, such as HIV, tuberculosis and malaria, as well as the increasing trends in non-communicable diseases, put a particularly high burden onto healthcare systems. These factors, coupled with suboptimal sanitation and water supply facilities, are reflected in the high maternal and neonatal mortality rates and the low life-expectancy estimates.

However, there is diversity in the socioeconomic conditions between individual countries in Africa, which can allow us to assume that the epidemiology of MRSA might also differ in different regions. It has not been well clarified, though, whether MRSA is more prevalent in African countries of low to medium human development index or vice-versa. In this review, we sought to assess the percentage of methicillin-resistance among *S. aureus* isolates in the region of Africa and whether this is associated with the human development index of each country according to the United Nations classification [25].

## Methods

### Literature Search

PubMed was systematically searched in January 2013. The following combined search term was applied: “(MRSA OR methicillin-resistant Staphylococcus aureus) AND (prevalence OR epidemiology OR surveillance) AND (Africa OR South Africa OR Western Africa OR Southern Africa OR Eastern Africa OR Central Africa OR Northern OR Africa South of the Sahara OR Africa)”. In addition, searches were performed in the World Wide Web by applying the following keywords to the Google search engine: “MRSA Africa”, “methicillin-resistant Staphylococcus aureus Africa”, “MRSA Africa (prevalence OR epidemiology OR surveillance)”, or “MRSA Africa resistance”. The Centers for Disease Control and Prevention (CDC), the World Health Organization (WHO) network, the African Field Epidemiology Network (AFENET), as well as the bibliographic references of all the included studies were hand-searched to identify additional potentially eligible studies. Articles published in English and French were only evaluated.

### Study Selection

Any article published after 2005, providing data for the percentage of MRSA among *Staphylococcus aureus* isolates collected from patients with different types of infection in Africa were considered eligible for inclusion in this review. Both clinical and microbiological (*in* *vitro*) studies that included isolates collected from patients meeting the afore-mentioned criteria were eligible. Studies reporting on neonates, children or adolescents were also included. Only studies reporting on non-duplicate isolates were included. Case reports and case series reporting on less than 100 *S. aureus* isolates were excluded.

### Data Extraction

The data extracted from each of the included studies consisted of the first author, year of publication, study design, country and period of the study, number of patients and/or isolates of *S. aureus* and the type of the culture specimen and of staphylococcal infection, the percentage of MRSA to the total *S. aureus* isolates, the percentage of the MRSA isolates that were positive for the Panton Valentine Leukocidin (PVL) toxin and the susceptibility of MRSA to the antibiotics tested in each study. The included studies were stratified primarily according to the Human Development Index (HDI) of the country that the study was performed in (defined by the United Nations as very high, high, medium and low HDI) [25].

### Focus of the Study

The focus of this review was, primarily, to assess the percentage of MRSA to the total *S. aureus* isolates in Africa, to which we refer to as “the prevalence of MRSA”. We also assessed the susceptibility of MRSA to different antibiotics and the percentage of the MRSA isolates that were positive for the PVL toxin. The susceptibility of the studied isolates was interpreted according to the criteria used in each study.

## Results

**Figure 1:**
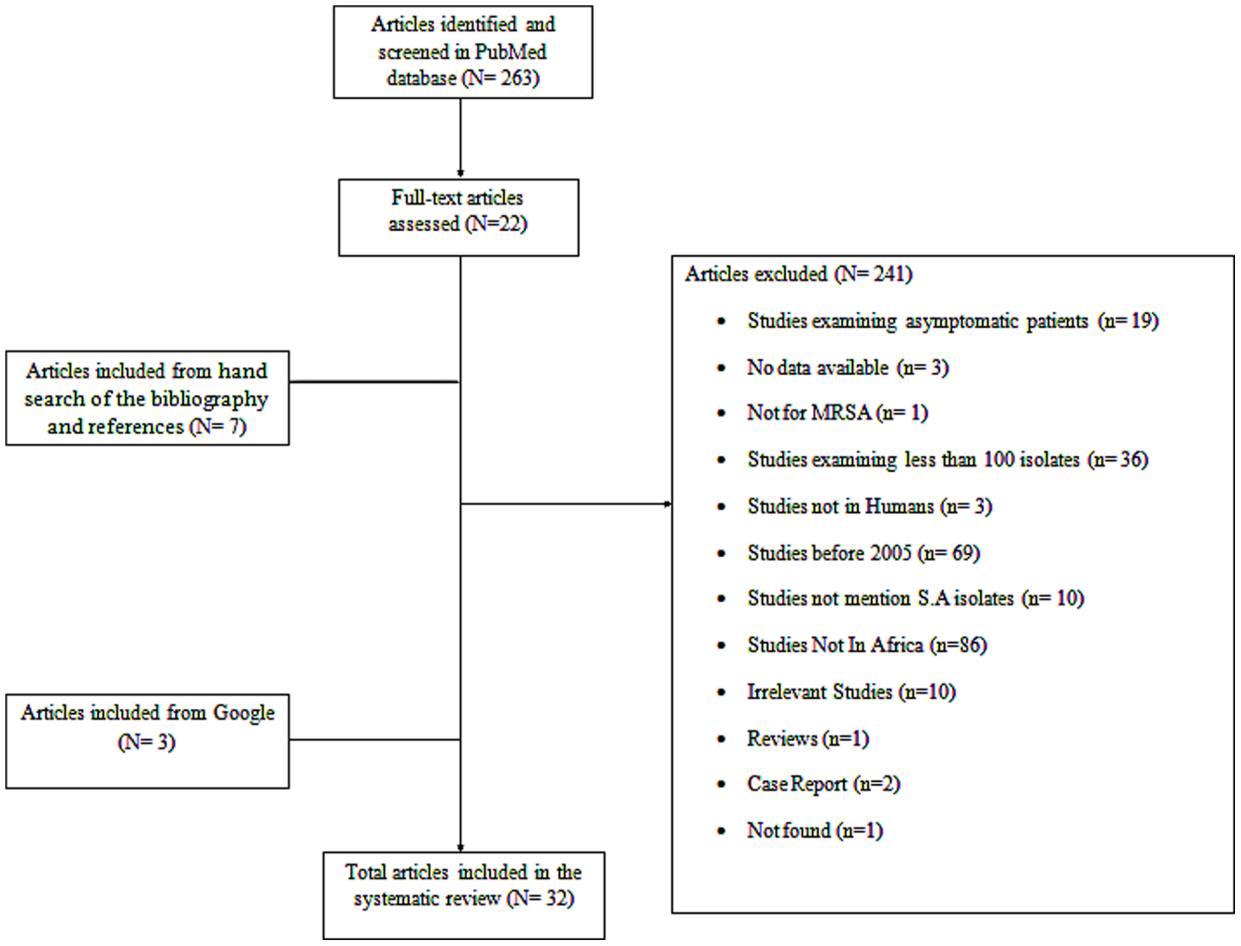
Graphical presentation of the process of selection of studies for inclusion in this review.

The process of identifying the relevant articles for inclusion in this review is depicted graphically in Figure 1. In total, 263 articles were evaluated. Of these, 241 articles were excluded due to the reasons presented in Figure 1, thus leaving 22 articles eligible for inclusion. Additionally, 3 more studies were retrieved from Google searches and 7 more studies were identified from hand-searching the bibliographic references of relevant studies. Finally, 32 studies were evaluated in this review [26], [27], [28], [29], [30], [31], [32], [33], [34], [35], [36], [37], [38], [39], [40], [41], [42], [43], [44], [45], [46], [47], [48], [49], [50], [51], [52], [53], [54], [55], [56], [57].

### High Human Development Index Countries

The data extracted regarding the prevalence of MRSA in high human development countries in Africa are presented in Table 1. Specifically, relevant to our review data were available for 2 countries, namely Tunisia and Libya. Regarding Tunisia, data derived from 7 studies referring to the period between 2002 and 2007 [28], [31], [32], [33], [42], [43], [53]. The first study was a retrospective one and referred mostly to pus specimens and blood cultures from one hospital during 2002–2003 [42]. The prevalence of MRSA was 16%. The PVL toxin was found in 2% of MRSA strains, which all exhibited glycopeptide intermediate susceptibility. Two prospective studies referred to the period between 2003 and 2004, and the prevalence of MRSA was similar, 13% and 12%, respectively. The first of these studies was conducted in 2 Tunisian hospitals as part of a multicenter study and assessed several types of clinical specimens, which were mostly isolated from ICU patients. The second of these studies was conducted in a university hospital and collected samples from hospitalized patients and patients attending the dermatology department [28], [32].

**Table 1:** Characteristics of the included studies regarding the epidemiology of MRSA in Africa.

Firstauthor,Year	Study design;country; period	Number of patientsand/or isolates	Culture Specimens	Type of infection	Percentage of MRSA among*S. aureus,* n/N (%)	PVL (+)MRSA,n/N (%)
**High Human Development Index Countries**						
**Mastouri,** **2006 ** [**42**]	Retrospective; Tunisia;June2002–December 2003	620 isolates *S. aureus*	Pus, blood, catheters, urine, pleuritic fluid, tracheal secretions, CSF fluid	NR	96/620 (16)	2/96 (2)
**Amazian,** a **2006 ** [**28**]	Prospective MC; Algeria,Tunisia; March2003–January 2004	Tunisia: 336 isolates*S. aureus*	Blood, subcutaneous,superficial suppurations,respiratory samples,intravascular device,urine, pressure sores	NR	42/336 (12)	NR
**Ben Saida,** **2005 ** [**32**]	Prospective; Tunisia;September 2003–March 2004	147 hospitalized or consultingin dermatology pts; 147isolates *S. aureus*	NR	NR	19/147 (13)	NR
**Borg,** a **2007 ** [**33**]	Retrospective Tunisia MC,(Algeria, Egypt, MoroccoMC); 2003 to 2005	Tunisia: 586 isolates*S. aureus*	Blood	Bacteremia	105/586 (18)	NR
**Ben Ayed,** **2006 ** [**31**]	Prospective; Tunisia;January 2004–June 2005	475 isolates *S. aureus*	Cutaneous pus, blood cultures, urine, materials, respiratory tract specimens, fluid punctions	Dermatology, surgery, pediatrics,gynecology neonatology clinics, ICU	57/475 (12)	NR
**Thabet,** **2008 ** [**53**]	Retrospective; Tunisia;January 2005–December 2006	375 isolates *S. aureus*	Blood, urine, respiratoryspecimens, catheters,cutaneous pus	NR	174/375 (46)	NR
**Mesrati,** **2010 ** [**43**]	Prospective; Tunisia;January 2005–July 2007.	141 pts (two of which wereeach carrying two distinctstrains); 143 *S. aureus* isolates	Pus, blood, fluid aspirates, pulmonary specimens, urine,medical devices, genital specimens	Cutaneous, deep-seated, abscesses,bacteremia, bone or joint, pleural-pulmonary, genitourinary, device-related	58/143 (41) CA: 22/77 (29) HA: 36/66 (55)	21/58 (36) CA:9/22 (41) HA:12/36 (33)
**Buzaid,** **2011 ** [**36**]	Retrospective; Libya;April–July 2007	200 *S. aureus* isolates	Pus, wound swabs, centralvenous line tips, endotrachealtube tips, blood cultures,urine catheter tips	Skin and soft tissue, surgical wounds,infections after invasive ICU procedures,chronic osteomyelitis, septic arthritis	62/200 (31)	NR
**Medium Human Development Index**						
**Perovic,** **2006 ** [**47**]	Retrospective MC; SouthAfrica; November1999 –October 2002	449 pts; 449 isolates*S. aureus*	Blood	Bacteremia	105/449 (23) CA: 21/204 (10) HA: 84/245 (34)	NR
**Shittu,** **2006 ** [**50**]	Retrospective 2 phaseMC; South Africa; March–August 2001and October2002–August 2003	227 isolates *S. aureus*	Pus (mostly), sputum, blood, otic fluid	NR	61/227 (27)	NR
**Groome,** **2012 ** [**41**]	Retrospective record review;South Africa; January 2005–December 2006	161 children	Blood	Community-acquiredbloodstream infections	63/161 (39)	NR
**Brink,** **2007 ** [**35**]	Prospective MC; South Africa;January 2006–June 2006	629 isolates *S. aureus*	Blood	Hospitalized pts withCA or HA infection	226/629 (36)	NR
**van** **Rensburg,** **2012 ** [**55**]	Retrospective surveillance;South Africa; July2007 –June 2011	13.746 *S. aureus* isolates	NR	NR	3298/13.746 (24)	NR
**Amazian,** a **2006 ** [**28**]	Prospective MC; Algeria,Tunisia; March2003–January 2004	Algeria: 203 isolates*S. aureus*	Blood, subcutaneous, superficialsuppurations, respiratory samples,intravascular device, urine, pressure sores	NR	72/203 (35)	NR
**Borg,** a **2007** [**33**]	Retrospective Algeria, Egypt,Morocco MC (Tunisia MC);2003 to 2005	1591 isolates *S. aureus*;Algeria 569	Blood	Bacteremia	Algeria 256/569 (45)	NR
**Antri,** **2010 ** [**29**]	Prospective microbiological;Algeria; April 2006 toDecember 2007	221 pts: 84 pts CA, 137 HA;221 isolates *S. aureus*	NR	Skin/soft-tissue, bone/joint,bacteremia, pneumonia, ENT or eye,meningitis urinary tract	99/221 (45) CA: 34/84 (40) HA: 65/137 (47)	CA: 30/84 (36);HA: 49/137 (36)
**Rebiahi,** **2011 ** [**49**]	Prospective; Algeria;April 2007–May 2009	287 pts; 220 *S. aureus*	Pus	Surgical wounds	165/220 (75)	NR
**Wood,** **2009 ** [**56**]	Cross-sectional; Botswana;2000– 2007	Adult, neonates, pediatric pts;582 *S. aureus*; 538 *S. aureus*with known susceptibilities	Blood	Bacteremia	239/538 (44)	NR
**Truong,** **2011 ** [**54**]	Retrospective cohort; Botswana;2000–2007	857 pediatric and adult pts;857 isolates *S. Aureus*	Pus, wound sites, eye, ear, throat,vagina, pleural, pericardial,joint fluid, brain abscesses	SSTIs and others not specified	194/857 (23)	NR
**Borg,** a **2007 ** [**33**]	Retrospective Algeria, Egypt,Morocco MC (Tunisia MC);2003 to 2005	1591 isolates *S. aureus*;Morocco 465	Blood	Bacteremia	Morocco 88/465 (19)	NR
**Elhamzaoui,** **2009 ** [**37**]	Prospective; Morocco; March2006–March 2008	461 isolates *S. aureus*	Blood culture, pus	Abscesses, ulcers, otitis, bacteremia,osteomyelitis, pulmonary infections	89/461 (19)	NR
**Ashour,** **2007 ** [**30**]	Prospective; Egypt; NR	Cancer pts; 140 isolates*S. aureus*	Urine, sputum, chest tube, bronchoalveolar lavage, pus, blood, throat swabs, and skininfection swabs	Various infection sites in cancerpatients (hematologic malignanciesand solid tumors)	114/140 (82)	NR
**Borg,** a **2007 ** [**33**]	Retrospective Algeria, Egypt, Morocco MC (Tunisia MC); 2003 to 2005	1591 isolates *S. aureus*;Egypt 557	Blood	Bacteremia	Egypt 290/557 (52)	NR

a Study refers to more than one countries

MRSA: Methicillin-resistant *S. aureus*; PVL: Panton Valentine Leukocidin; MC: Multicenter; NR: not reported; NE: non evaluable; CA: community-acquired; HA: hospital-acquired; CSF: cerebrospinal fluid; ICU: Intensive Care Unit; ENT; ear, nose and throat; SSTIs: skin and soft tissue infections.

A more representative and clinically relevant study in Tunisia was part of the Antibiotic Resistance Surveillance and Control in the Mediterranean Region (ARMed) project and referred to the period between 2003 and 2005 [33]. The prevalence of MRSA among blood cultures in 4 Tunisian hospitals was 18%. In another, prospective study conducted in 1 hospital in 2004–2005, which analyzed mostly samples of cutaneous pus and blood, the prevalence of MRSA was 12% [31]. In the remaining 2 studies, the one done in 2005–2006 and the other in 2005–2007, each conducted in a single hospital, the prevalence of MRSA rose to 46% and 41%, respectively [43], [53]. The clinical samples were mostly blood cultures and cutaneous pus in the former study, and cutaneous pus, blood cultures and samples from deep seated infections, in the latter study. Among the 58 (41%) of MRSA isolates from one of the above-mentioned studies, (29%) were community-acquired and (55%) were hospital-acquired. Forty-one percent of the community- acquired isolates and 33% of the hospital-acquired isolates were PVL toxin positive.

One study has reported on the prevalence of MRSA in Libya [36]. This was a retrospective study, which assessed 200 isolates in a surgical and trauma hospital for a period of 4 months during 2007. The prevalence of MRSA was 31%.

### Medium Human Development Index Countries

The data extracted regarding the prevalence of MRSA in medium human development countries in Africa are presented in Table 1. Specifically, relevant data to our review were available for 5 countries, namely South Africa, Algeria, Botswana, Morocco and Egypt. All studies included more than 200 patients, except from one study in Egypt that included 140 patients. Regarding South Africa, data derived from 5 studies. The first study was retrospective in nature and assessed 449 bacteremic patients in 1999–2002 in two academic hospitals [47]. The prevalence of MRSA was 23%. Community-acquired MRSA accounted for 20% hospital-acquired MRSA accounted for 80% of the total MRSA isolates. Another retrospective study was conducted in 14 health institutions in 2001–2003 [50]. Pus from wound specimens accounted for most of the clinical samples. The prevalence of MRSA was 27%.

A prospective nationwide study from 12 laboratories of 7 private pathology practices in South Africa was done over a 6-month period in 2006 and assessed blood isolates [35]. The prevalence of MRSA was 36%. A retrospective study assessed children with community-acquired bacteremia through 2005–2006 in a setting with high prevalence of HIV infection [41]. The prevalence of MRSA in this population was 39%. The most recent study from South Africa was retrospective in nature and referred to a period between 2007 and 2011 [55]. A large number of isolates, 13.746 in total, were collected from three tertiary- and two secondary-level public hospitals. The prevalence of MRSA was 24%.

Regarding Algeria, available data derived from four studies. The first study was conducted in 2003–2004 in four hospitals, reported a prevalence of MRSA of 35% [28]. Half of the specimens were collected from subcutaneous punctures, 17% from urine and 11% from blood. One third of the MRSA isolates were collected in maternity units. The second study, which was part of the Antibiotic Resistance Surveillance and Control in the Mediterranean Region (ARMed) project, assessed bacteremic episodes during 2003–2005 in 567 patients, in 23 hospitals [33]. The prevalence of MRSA was 45%. The third study, which was conducted in 2006–2007, provided data for community and hospital acquired MRSA, regarding mostly skin and soft tissue infections [29]. The prevalence of MRSA was 40% and 47%, respectively, while the prevalence of MRSA among all the isolates was 45%. In the same study the carriage of the PVL toxin was similar (36%) in both community and hospital acquired MRSA. The fourth study, which was conducted in 2007–2009, reported on MRSA isolated from pus in surgical patients in a single hospital [49]. The prevalence of MRSA was 75%.

In Botswana, 2 studies were identified as relevant to this review, which both referred to the period between 2000 and 2007 [54], [56]. In the first study, which included neonates, children and adults, the prevalence of MRSA was 23%. The prevalence of MRSA was 18% specifically for skin and soft tissue infections in patients13–49 years of age, who accounted for almost two thirds of the study population. In the second study, which included neonates, children and adults, the prevalence of MRSA was 44%, and it was higher in neonates and adults than children.

In Morocco, according to 2 included studies, which referred to the period of 2003–2005 and 2006–2008, the prevalence of MRSA was 19% in both studies. The study reporting for 2003–2005 was part of the Antibiotic Resistance Surveillance and Control in the Mediterranean Region (ARMed) project [33]. The study included bacteremic patients in 3 Moroccan hospitals that had a capture population of about 4% of the total country population. The second study was conducted during 2006–2008 in two Moroccan teaching hospitals, included patients with bacteremia and pyogenic infections [37].

Regarding Egypt, we identified two studies as eligible for inclusion in this review [30], [33]. The first study, which did not report on the period of collection of the isolates, included cancer patients only, and the prevalence of MRSA was 82% [30]. The second study, referring to 2003–2005, was done in nine hospitals as part of the Antibiotic Resistance Surveillance and Control in the Mediterranean Region (ARMed) project, with a capture population of 17% of the total country population [33]. The prevalence of MRSA among *S. aureus* blood cultures was 52%, exhibiting an increasing trend during the 3-year period of the study.

### Low Human Development Index Countries

The data extracted regarding the prevalence of MRSA in low human development countries in Africa are presented in Table 2. Specifically, 13 studies were evaluated [26], [27], [34], [38], [39], [40], [44], [45], [46], [48], [51], [52], [57]. Out of these, 7 referred to Nigeria, and 1 to each of the following countries: Tanzania, Ethiopia, Ivory Coast, Eritrea, and Madagascar. There was also 1 multinational study with data from Madagascar, Cameroon, Senegal and Niger.

**Table 2:** Characteristics and outcomes of the included studies

First author,Year	Study design; country; period	Number of patients and/or isolates	Culture Specimens	Type of infection	Percentage ofMRSA among *S. aureus* n/N (%)	PVL (+) MRSA n/N (%)
**Low Human Development Index**						
**Adesida, 2005** [**7**]	Retrospective MC; Nigeria; August 1998–June 2002	276 isolates*S. aureus*	Variety of clinicalmaterials, MRSA:Aspirate, wound,amniotic fluid	Invasive infections; MRSA: Recurrent septic arthritis, preterm contraction,chronic osteomyelitis	26/276 (9)	NR
**Taiwo, 2005** [**51**]	Retrospective; Nigeria; January–December 2001	141 isolates*S. aureus*	SSTIs followingsurgery or trauma	NR	45/141 (32)	NR
**Ghebremedhin,** **2009 ** [**40**]	Prospective MC; Nigeria;2006–2007	1,300 adultand pediatricpts; 346isolates *S.* *aureus*	Wounds, corneal,conjunctival,auricular, genital,nasal swabs	Conjunctivitis, cataracts, otitis,pyomyositis, cellulitis, burns, UTI,trauma, fracture, posttraumatic/postsurgical wounds, diabetic foot, skin infections	70/346 (20) HA:37/70 (53) CA:33/70 (47)	33/70 (47)all CA-MRSA
**Fayomi, 2011** [**38**]	Prospective; Nigeria;January–December 2009	158 isolates*S. aureus*	Pus, aspirates,sputum, throatswabs, urine, CSF,vaginal swabs,semen, blood	NR	49/158 (31)	NR
**Nwankwo,** **2011 ** [**46**]	Retrospective; Nigeria;January–December 2009	150 isolates*S. aureus*	Wounds, blood,umbilical cord,urine, ENT, abscess,catheter tips, pleuralaspirate and skin swab	NR	16/150 (11)	NR
**Terry Alli,** **2012 ** [**52**]	Retrospective MC;Nigeria; NR	116 isolates*S. aureus*	Wound, eye, ear,urine, endocervical,urine, aspirate andblood	NR	48/116 (41) CA:3/48 (7) HA:45/48 (93)	28/116(24)
**Ghamba, 2012** [**39**]	Prospective; Nigeria; NR	150 pts; 150isolates *S.* *aureus*; (2healthysubjectsexcluded)	Urine, vaginal,wound, urethral,seminal, sputum,endocervical, earand nasal swabs	NR	42/148 (28) CA:18/42 (43) HA:24/42 (57)	NR
**Randrianirina,** **2007 ** [**48**]	Retrospective; Madagascar; January 2001–December 2005	574 isolates*S. aureus*; CA506 HA 68	CA: genital tract,pus, urinary tract,respiratory tract HA:surgical wounds,cutaneous pus, blood	Community and hospital acquired infections	36/574 (6) CA:33/506 (7) HA:3/68 (4)	NR
**Breurec, 2010** [**34**]	Prospective; Madagascar, Niger, Senegal, Cameroon; January 2007–March 2008	542 isolates*S. aureus*	Pus, blood, urine,pulmonary secretions	SSTIs, surgical wounds, bacteremia/septicemia, urinary/genital tract,osteomyelitis/myositis, pulmonaryinfections	86/542 (16)	17/86 (20)(13/17HA)
**Abera, 2008** [**26**]	Prospective cross sectional; Ethiopia; April–June 2006	151 inpts, 70outpts; 162isolates*S. aureus*	Surgical wound,ENT swabs, urine	NR	89/162 (55)	NR
**Zinzendorf,** **2008 ** [**57**]	Retrospective MC; IvoryCoast; NR	Adults andpediatric pts;180 isolates*S. aureus*	NR	NR	70/180 (39)	NR
**Mshana, 2009** [**44**]	Prospective; Tanzania.;April–July 2008	160 isolates*S. aureus*	Pus, wound swabsand aspirates	NR	26/160 (16)	NR
**Naik, 2009** [**45**]	Retrospective; Eritrea; NR	278 isolates*S. aureus*	Pus, ear discharge	NR	26/278 (9)	NR

a: Study refers to more than one countries.

MRSA: Methicillin-resistant *S. aureus*; PVL: Panton Valentine Leukocidin; MC: Multicenter; NR: not reported; NE: non evaluable; CA: community-acquired; HA: hospital-acquired; SSTIs: skin and soft tissue infections; UTI: urinary tract infection; CSF: cerebrospinal fluid; ENT; ear, nose and throat.

In Nigeria, one study was conducted in 10 hospitals in the north-central part during 1998–2002, and reported a prevalence of MRSA of 9% [27]. Samples were collected from several clinical sites and infections. In another study, referring to 2002 for the south-west part of the study, the prevalence of MRSA was 32% [51]. Samples were collected from patients with skin and soft tissue infections and infections following surgery and trauma. Another study was conducted during 2006–2007 in 2 hospitals in the south-west part of the country, assessing mostly wound samples from adult, pediatric, and neonatology patients [40]. The prevalence of MRSA was 20%. Forty-seven percent and 53% of the isolates were community-and hospital-acquired, respectively. For community-acquired isolates, otitis, conjunctivitis and skin infections were the commonest infections.

In a retrospective study, conducted during 2009 in the northwest part of Nigeria, including several clinical specimens, the prevalence of MRSA was 11% [46]. In a prospective study, which was conducted also in 2009, in the south-west part of Nigeria, and assessed *S. aureus* isolates from various types of clinical specimens, the prevalence of MRSA was 31% [38]. Another study in the south-west part of Nigeria, which did not mention the exact study period, reported a prevalence of MRSA of 41% for among several types of samples [52]. Most of these MRSA isolates (93%) were hospital-acquired. The PVL toxin was detected in 24% of the MRSA isolates. Finally, a study conducted in the north-eastern part of the country, which did not mention the study period, reported a prevalence of MRSA of 28% for various clinical specimens, with 43% of MRSA originating from outpatients and 57% from inpatients [39].

A retrospective study from Madagascar reported on a 4-year period (2001–2005) [48]. Most of the analyzed isolates were community-acquired (88%) and originated from genital and urinary specimens and pus. The overall prevalence of MRSA was 6%, while for the community- and hospital-acquired infections the prevalence of MRSA was 7% and 4%, respectively. In the prospective multinational study from Madagascar, Senegal, Cameroon and Niger, which was performed during 2007–2008, the prevalence of MRSA among specimens collected from skin and soft tissue infections (SSTIs) and surgical site infections was 16% [34]. The PVL toxin was positive in 20% of isolates, of which 76% were hospital-acquired.

A prospective study from a teaching hospital in Tanzania, which serves 13 million people, reported a prevalence of MRSA of 16% for a 4-month period in 2008 [44]. Specimens were mostly collected from surgical patients. A prospective study in both outpatients and inpatients of Ethiopia, which was performed during a 3-month period of 2006, reported a prevalence of MRSA of 55% [26]. Most of the clinical specimens were collected from surgical wounds. A retrospective study, in 3 teaching hospitals in Ivory Coast among adult and pediatric patients, reported a prevalence of 39% for MRSA [57]. In Eritrea, a retrospective study, which did not mention the exact study period, reported a prevalence of 9% of MRSA among pus specimens [45].

### Temporal Trend in the Prevalence of the MRSA

The temporal trends in the prevalence of MRSA in the high, medium and low human development index countries are presented in Table 3. An increase in the prevalence of MRSA between earlier and later studies during the last ten years could be noted for Tunisia and Algeria, while the opposite could hold true for South Africa. In Nigeria, the data in regard were split to specific regions, while for the remaining countries there were poor relevant comparative data.

**Table 3:** Summary table of the temporal trends in the percentage of MRSA among *S. aureus* in different countries.

High Human Development Index Countries		
First author, year	Country; Period	Percentage of MRSA among *S. aureus,* n/N (%)
**Mastouri, ** [**42**] ** 2006**	**Tunisia**; June 2002–December 2003	96/620 **(16)**
**Amazian, ** [**28**] ** 2006^a^**	**Tunisia**, Algeria; March 2003–January 2004	42/336 **(12)**
**Ben Saida, ** [**32**] ** 2005**	**Tunisia**; September 2003–March 2004	19/147 **(13)**
**Borg, ** [**33**] ** 2007^a^**	**Tunisia**, Algeria, Egypt, Morocco; 2003 to 2005	105/586 **(18)**
**Ben Ayed, ** [**31**] ** 2006**	**Tunisia**; January 2004–June 2005	57/475 **(12)**
**Thabet, ** [**53**] ** 2008**	**Tunisia**; January 2005–December 2006	174/375 **(46)**
**Mesrati, ** [**43**] ** 2010**	**Tunisia**; January 2005–July 2007.	58/143 **(41)**, CA: 22/77 (29), HA:36/66 (55)
**Buzaid, ** [**36**] ** 2011**	**Libya**; April–July 2007	62/200 **(31)**
**Medium Human Development Index Countries**		
**First author, year**	**Country; Period**	**Percentage of MRSA among ** ***S. aureus,*** ** n/N (%)**
**Perovic, ** [**47**] ** 2006**	**South Africa**; November 1999–October 2002	105/449 **(23)**, CA: 21/105 (20), HA: 84/105 (80)
**Shittu, ** [**50**] ** 2006**	**South Africa**; March–August 2001 andOctober 2002–August 2003	61/227 **(27)**
**Groome, ** [**41**] ** 2012**	**South Africa**; January 2005–December 2006	63/161 **(39)**
**Brink, ** [**35**] ** 2007**	**South Africa**; January 2006–June 2006	226/629 **(36)**
**van Rensburg, ** [**55**] ** 2012**	**South Africa**; July 2007–June 2011	3298/13.746 **(24)**
**Amazian, ** [**28**] ** 2006^a^**	**Algeria**, Tunisia; March 2003–January 2004	72/203 **(35)**
**Borg, ** [**33**] ** 2007^a^**	**Algeria**, Egypt, Morocco MC (Tunisia MC);2003 to 2005	256/569 **(45)**
**Antri, ** [**29**] ** 2011**	**Algeria**; April 2006 to December 2007	99/221 **(45)**, CA: 34/84 (40), HA: 65/137 (47)
**Rebiahi, ** [**49**] ** 2011**	**Algeria**; April 2007–May 2009	165/220 **(75)**
**Wood, ** [**56**] ** 2009**	**Botswana**; 2000–2007	239/538 **(44)**
**Truong, ** [**54**] ** 2011**	**Botswana**; 2000–2007	194/857 **(23)**
**Borg, ** [**33**] ** 2007^a^**	Algeria, Egypt, **Morocco** MC (Tunisia MC);2003 to 2005	88/465 **(19)**
**Elhamzaoui, ** [**37**] ** 2009**	**Morocco**; March 2006–March 2008	89/461 **(19)**
**Ashour, ** [**30**] ** 2007**	**Egypt**; NR	114/140 **(82)**
**Borg, ** [**33**] ** 2007^a^**	Algeria, **Egypt**, Morocco MC (Tunisia MC);2003 to 2005	290/557 **(52)**
**Low human Development Index Countries**		
**First author, year**	**Country; Period**	**Percentage of MRSA among ** ***S. aureus,*** ** n/N (%)**
**Adesida, ** [**27**] ** 2005**	**Nigeria NC**; August 1998–June 2002	26/276 **(9)**
**Taiwo, ** [**51**] ** 2005**	**Nigeria SW**; January–December 2001	45/141 **(32)**
**Ghebremedhin, ** [**40**] ** 2009**	**Nigeria SW**; 2006–2007	70/346 **(20)**, CA: 33/70 (47), HA: 37/70 (53)
**Fayomi, ** [**38**] ** 2011**	**Nigeria SW**; January–December 2009	49/158 **(31)**
**Nwankwo, ** [**46**] ** 2011**	**Nigeria NW**; January–December 2009	16/150 **(11)**
**Terry Alli, ** [**52**] ** 2012**	**Nigeria SW**; NR	48/116 **(41)**, CA: 3/48 (7), HA: 45/48 (93)
**Ghamba, ** [**39**] ** 2012**	**Nigeria NE**; NR	42/148 **(28)**, CA: 18/42 (43), HA: 24/42 (57)
**Randrianirina, ** [**48**] ** 2007**	**Madagascar**; January 2001–December 2005	36/574 **(6)**, CA: 33/506 (7), HA: 3/68 (4)
**Breurec, ** [**34**] ** 2010**	**Madagascar**, **Niger**, **Senegal**, **Cameroon**;January 2007–March 2008	86/542 **(16)**
**Abera, ** [**26**] ** 2008**	**Ethiopia**; April–June 2006	89/162 **(55)**
**Zinzendorf, ** [**57**] ** 2008**	**Ivory Coast**; NR	70/180 **(39)**
**Mshana, ** [**44**] ** 2009**	**Tanzania**; April–July 2008	26/160 **(16)**
**Naik, ** [**45**] ** 2009**	**Eritrea**; NR	26/278 **(9)**

MRSA: Methicillin-resistant *S. aureus*; CA: community-acquired; HA: hospital-acquired; MC: multicenter; NC: north-central; SW: south-west; NW: north-west; NE: north-east; NR: not reported

### Community- and Hospital-acquired MRSA

Data on the prevalence of MRSA among community or hospital acquired *S. aureus* was reported in 4 studies [29], [43], [47], [48]. In 2 of these studies, the prevalence of MRSA was lower in the community-acquired *S. aureus,* while in the remaining two studies no significant differences could be observed, although in one of them the number of hospital-acquired isolates is rather low.

### Susceptibility of MRSA to Various Antibiotics

Detailed data on the susceptibility of the MRSA isolates collected in each of the included studies to the various antibiotics tested are presented in Table 4. Considering all of the included studies together, the susceptibility of MRSA isolates in Africa to various antibiotics varied as follows: rifampicin 22%–100%, gentamicin 0–100%, vancomycin 82–100%, ofloxacin 40–100%, ciprofloxacin 25–100%, chloramphenicol 0–100%, cotrimoxazole 0–100%, erythromycin 0–100%, fusidic acid 33–100%, tetracycline 0–100%, clindamycin 18–100%, teicoplanin 93–100%, fosfomycin 84–99% and linezolid 85–100%. The relatively high number of MRSA isolates that were reported as non-susceptible to glycopeptides or linezolid in some studies should be interpreted with caution. In two of these studies, the susceptibility to vancomycin was determined with the disk diffusion method or with an automated system, which are not considered robust in this regard. None of these studies assessed for the presence of any of the vancomycin resistance genes.

**Table 4:** Susceptibility to different antibiotics of MRSA isolates collected in African countries of high, medium, and low human development index.

HIGH																			
COUNTRY	AUTHOR	PERIOD	N	RIF	GEN	VAN	OFX	CIP	CLH	SXT	ERY	FA	TET	CLI	TEC	FOF	GISA	AMK	LZD
				**n (%)**															
**Libya**	**Buzaid, 2011 ** [**35**]	**April–July 2007**	62			51 (82)^a^		41 (66)	38 (61)		33 (53)	36 (58)							
**Tunisia**	**Mastouri, 2006 ** [**41**]	**June 2002–December 2003**	96	94(98)	79(82)	95 (99)^C^	57 (59)		93 (97)	84 (87)	49 (51)	39 (41)	33 (34)		94 (98)	89 (93)	2 (2)		
	**Ben Saida, 2005 ** [**31**]	**September 2003–March 2004**	19	12(63)	0 (0)		14 (74)			14 (74)	11 (58)	18 (95)	3 (16)			16 (84)			
	**Mesrati, 2010 ** [**42**]	**January 2005–July 2007**	PVL-CA: 9PVL- HA: 12	PVL-CA9 (100)PVL- HA12 (100)	PVL-CA9 (100)PVL-HA12 (100)	PVL-CA9 (100) PVL-HA12 (100)	PVL-CA9 (100) PVL-HA11 (92)		PVL-CA9 (100)PVL-HA12 (100)	PVL-CA9 (100) PVL-HA12 (100)	PVL-CA6 (67) PVL-HA7 (58)		PVL-CA0 (0)PVL-HA4 (33)	PVL-CA9 (100) PVL-HA11 (92)	PVL-CA9 (100)PVL-HA12 (100)				
**MEDIUM**																			
**COUNTRY**	**AUTHOR**	**PERIOD**		**RIF**	**GEN**	**VAN**	**OFX**	**CIP**	**CLH**	**SXT**	**ERY**	**FA**	**TET**	**CLI**	**TEC**	**FOF**	**GISA**	**AMK**	**LZD**
**South Africa**	**Shittu, 2006 ** [**49**]	**March–August 2001 and October 2002–August 2003**	61	16 (26)	2 (3)	61 (100)	50 (82)		51 (84)	9 (15)	11 (18)	61 (100)	6 (10)	11 (18)	61 (100)				
	**Groome, 2012 ** [**40**]	**January 2005–December 2006**		Children with HIV: (22)				>(60)		Children with HIV: (0)		>(60)						>(60)	
	**Brink, 2007 ** [**34**]	**January–June 2006**	629	560 (89)	554 (88)	629 (100)				447 (71)		610 (97)			629 (100)				629 (100)
	**van Rensburg, 2012 ** [**54**]	**July 2007–June 2011**	3298	1866 (57)															
**Algeria**	**Rebiahi, 2011 ** [**48**]	**April 2007–May 2009**	165		115 (70)	162 (98)^d^					73 (44)			145 (88)		154 (93)			
**Morocco**	**Elhamzaoui, 2009 ** [**36**]	**March 2006–March 2008**	89	62 (70)	40 (45)	89 (100)	36 (40)			42 (47)	36 (40)	29 (33)			83 (93)	84 (94)			
**Egypt**	**Ashour, 2007 ** [**29**]	**NR**	114			96 (84)^b^													97 (85)^b^

**LOW**																			
**COUNTRY**	**AUTHOR**	**PERIOD**		**RIF**	**GEN**	**VAN**	**OFX**	**CIP**	**CLH**	**SXT**	**ERY**	**FA**	**TET**	**CLI**	**TEC**	**FOF**	**GISA**	**AMK**	**LZD**
**Nigeria**	**Taiwo, 2005 ** [**50**]	**January–December 2001**	45			45 (100)													
	**Ghebremedhin, 2009 ** [**39**]	**2006–2007**	33	CA-MRSA33 (100)	CA-MRSA 33 (100)	CA-MRSA 33 (100)		CA-MRSA 33 (100)		CA-MRSA 1 (3)	CA-MRSA 33 (100)	CA-MRSA 33 (100)	CA-MRSA 33 (100)		CA-MRSA 33 (100)				CA-MRSA 33 (100)
	**Nwankwo, 2011 ** [**45**]	**January–December 2009**	16		0 (0)	16 (100)	11 (69)	9 (56)	0 (0)	0 (0)	0 (0)		0 (0)						
	**Fayomi, 2011 ** [**37**]	**January–December 2009**	49		12 (24)	49 (100)		43 (88)		4 (8)	15 (31)			37 (76)					
	**Ghamba, 2012 ** [**38**]	**NR**	42		38 (90)		33 (79)	37 (88)	17 (40)		21 (50)								
**Tanzania**	**Mshana, 2009 ** [**43**]	**April–July 2008**	26			26 (100)		15 (58)	17 (65)	2 (8)	8 (31)		9 (35)	9 (35)					
**Madagascar**	**Randrianirina, 2007 ** [**47**]	**January 2001–December 2005**	36	31 (86)	32 (89)	36 (100)		31 (86)	28 (78)	22 (61)	24 (67)	29 (81)	9 (25)		36 (100)	35 (97)			
**Madagascar, Niger, Senegal, Cameroon**	**Breurec, 2010 ** [**33**]	**January 2007–March 2008**	76	47 (62)	38 (50)					21 (28)	41 (54)	64 (84)	9 (12)			75 (99)			
**Ethiopia**	**Abera, 2008 ** [**25**]	**April–June 2006**	89		26 (29)	89 (100)		22 (25)			23 (26)		9 (10)						
**Eritrea**	**Naik, 2009 ** [**44**]	**NR**	26		14 (54)			24 (92)	19 (73)	20 (77)	19 (73)		3 (12)						

a Vancomycin susceptibility was determined with a disk diffusion test.

b Vancomycin and linezolid susceptibility was determined with automated system testing.

c 1 Isolate had a vancomycin minimum inhibitory concentration of 6 mg/l determined with the E-test method.

d 3 Isolates had vancomycin minimum inhibitory concentrations between 16 and 128 mg/L determined with the agar dilution method.

ERY: erythromycin; LIN: lincosamide; TET: tetracycline; RIF: rifampicin; SXT: trimethoprim-sulfamethoxazole; CLH: chloramphenicol; GEN: gentamicin; OFX: ofloxacin; CIP: ciprofloxacin; VAN: vancomycin; TEC: teicoplanin; FA: fusidic acid; FOF: fosfomycin; LZD: linezolid; AMK: amikacin; GISA: glycopeptide intermediate S. aureus

## Discussion

The available evidence regarding the prevalence of methicillin resistance among *S. aureus* isolates (“prevalence of MRSA”) collected in the African countries in different relevant studies yields variable findings, making the extrapolation of definitive relevant conclusions rather difficult. Certainly though, MRSA poses a visible threat in many African countries. The spread of MRSA in the African region can be worrisome, since there might be relatively limited availability of modern antibiotics effective against hospital-associated MRSA, like linezolid and daptomycin, in most of this part of the world. Furthermore, the implementation of infection control measures and the wide spread of HIV infection and tuberculosis, particularly in the sub-Saharan area, amplify the difficulty of dealing with the MRSA epidemic in Africa.

In an attempt to summarize the data evaluated in this review, we observe that in most of the high and medium human development index countries analyzed, the prevalence of MRSA gradually rose during the first years of the new millennium. The most pronounced increase was observed in Tunisia, with an increase to 41–46% after 2005, compared with a prevalence of 12%–18% in the years before. Yet, in South Africa, the prevalence of MRSA decreased from 36% in 2006 to 24% during 2007–2011, probably due to the implementation of effective infection control policies. In Botswana, the prevalence of MRSA varied between 23% and 44% for the period of 2000–2007, in 2 relevant studies. In Algeria and Egypt, according to a multicenter study, the prevalence of MRSA between 2003–2005 was 45% and 52%, respectively. Morocco is the only country where a low prevalence of MRSA seems to have been stabilized during 2003–2008. However, for most of the countries evaluated, the data for the prevalence of MRSA in different calendar years come from different studies, while the majority of these studies are single-centered. There are therefore many potential differences that should be taken into consideration, such as the country regions of reference, the type of the patient population examined, and the microbiological and sampling methods used.

Among the low human development index countries, Madagascar, Senegal, Cameroon, Niger, Eritrea and Tanzania have sustainably had a low prevalence of MRSA, varying from 6% to 16%, during 2001–2009 (according to data derived from individual studies). Moremi et al, and Blomberg et al, report similar results regarding the prevalence of MRSA in Tanzania [58], [59]. This could hypothetically be attributed to underutilization of antibiotics in the poorer compared with the wealthier countries, leading to a comparatively lower selection pressure for MRSA [48]. On the other hand, in Nigeria the prevalence of MRSA appears to be different in the northern compared with the southern part of the country. For the northern part of Nigeria, the prevalence of MRSA varied from 9% in north-central to 28% in north-east, while in the south-west part of the country it was between 20% and 41%. To notice, Nigeria is geophysically divided in the northern part with the savanna and the Sahel steppe and in a southern part where dense vegetation exists. In Ethiopia and in the Ivory Coast, the prevalence of MRSA was 55% and 39%, respectively (data from individual studies).

With cautious estimates, where specific relevant data were available, it seems that the majority of the MRSA isolates were hospital-acquired for most of the included studies. This finding does not exclude the possibility that there is a considerable reservoir of community-acquired MRSA in Africa. It could be attributed to the fact that hospital-acquired isolates were more easily recorded in the included studies.

The susceptibility of MRSA to various antibiotics varied among the included studies. This can represent between studies differences in the type of the studied population; for example some studies included hospitalized cancer patients or patients with HIV infection. The observed differences in antibiotic susceptibility of MRSA in Africa could mainly be attributed to the prevalent use of certain antibiotics in some countries, either due to availability and cost-effectiveness issues or due to administration of antibiotics for other illnesses such as the use of rifampicin for treating tuberculosis. In studies that included many patients previously exposed to antibiotics, it is likely that MRSA isolates had been selected. Conversely, in a study evaluating the characteristics of *S. aureus* isolates collected from remote Gabonese Babongo Pygmies, none of the 34 isolates was resistant to methicillin, while 61,8% of the isolates remained susceptible to penicillin [60].

The prevalence of MRSA in Africa during the last decade, as evidenced in this review, appears to be higher compared with that before 2000, at least for certain regions [61]. In the great majority of the studies included in this review the prevalence of MRSA was either between 25% and 50%, or less than 25%. These estimates must be compared with those from other parts of the world. Specifically, in Asian countries the overall prevalence of MRSA reaches 39% [62]. In European countries it varies between <1% in Iceland, Belgium, Netherlands and >50% in Portugal and Romania according to ECDC estimates for 2011. Regarding the Mediterranean European countries, the prevalence of MRSA varies from 25%–50% in Italy, Greece, Cyprus, Turkey to 59% in Malta and 56% in Israel [63]. Likewise, in the northern countries of Africa drenched by the Mediterranean sea, the prevalence of MRSA varies from 31% in Libya and 45% in Algeria and Tunisia to 52% in Egypt, with Morocco (the western of these countries) being the only one exhibiting a prevalence of 19%. Regarding the USA, the prevalence of MRSA appears to have been declining from 2005 through 2010, with a 51% estimate in 2009 [64].

Our approach to systematically evaluate the published literature on the prevalence of MRSA in Africa should be viewed in light of some potential limitations. As it is common for this type of reviews, we have included studies done in various settings using different methodologies regarding the patient selection criteria and the microbiological methods. Differences in the methods used can influence the findings on the prevalence of methicillin resistance among the *S. aureus* isolates. The great majority of the studies relied on phenotypic rather than molecular tests for the detection of MRSA. If molecular methods are used for confirmation of the MRSA isolates, the percentage of methicillin-resistance can be expected to be lower. Moreover, some studies used the oxacillin disk diffusion test for detecting MRSA which is considered less accurate than the cefoxitin disk diffusion test in this regard. The type of agar used can also influence the above findings.

We also set a pre-specified criterion to include only those studies that reported on at least 100 *S. aureus* isolates from patients with an infection, so as to select those studies that could provide more information that could be clinically relevant. Inevitably, some studies with a methodology of higher quality were not included. For example, a study in Gabon evaluated the molecular diversity and the virulence factors of prospectively collected *S. aureus* isolates between 2008 and 2010 [65]. Differences were observed between the isolates from asymptomatic carriers and those from patients with an infection. The percentage of methicillin resistance was 3.7% in the 163 isolates from carriers and 11,1% in the 44 isolates from patients with an infection. Another study examined the molecular and resistance characteristics of staphylococcal clinical isolates collected in 8 hospitals in Nigeria in 2010 [66]. The percentage of methicillin resistance (confirmed with detection of the *mecA* gene) in 51 *S. aureus* isolates was 29,4%. Differences in the clonal characteristics of MRSA were noted between North-East and South-West Nigeria.

In conclusion, the greatest amount of data for the prevalence of methicillin resistance in clinical *S. aureus* isolates over the last decade in Africa derives from the countries in the Mediterranean basin, South Africa, and Nigeria. According to this data, as well as data contributed by other countries, the burden of MRSA in Africa is not negligible. The prevalence of MRSA was at the level of 25–50% for many countries, while it was lower than 25% for some other countries. Regional differences in antibiotic availability and consumption or in the spread of HIV and tuberculosis could account for differences in MRSA prevalence between sub-Saharan countries. No decreasing trend in the prevalence of MRSA in individual countries over the study period could be noted, except possibly for South Africa. The spread of MRSA in Africa must be taken into consideration in the global battle against antimicrobial resistance.
